# Differences in the structural features of atypical adenomatous hyperplasia and low-grade prostatic adenocarcinoma

**DOI:** 10.4103/0970-1591.40610

**Published:** 2008

**Authors:** Ahmet Midi, Tülay Tecimer, Süheyla Bozkurt, Naziye Özkan

**Affiliations:** Department of Pathology, Marmara University Hospital, Istanbul, Turkey

**Keywords:** Adenosis, cancer, hyperplasia, low grade, proliferations, prostate, small glandular

## Abstract

**Aim:**

Atypical adenomatous hyperplasia (AAH) is a small glandular proliferation that has histological similarities with Gleason grade 1 and 2 prostatic adenocarcinoma (PACG1,2). There are no distinct histomorphological criteria distinguishing these two lesions from each other and other small glandular proliferations. Because treatment approaches are different for these lesions, it is necessary to determine histological criteria. The aim of this study is to review the histological features of these two lesions and to define new histological criteria distinguishing AAH from PACG1,2. We, therefore, assessed 18 anatomical and structural parameters.

**Materials and Methods:**

We found 11 AAH (22 foci) and 15 PACG1,2 (22 foci) cases in 105 radical prostatectomy specimens. Basal cell-specific antikeratin was applied to these lesions. We assumed that PACG1,2 lesions did have not basal cells and we grouped the lesions as AAH and PACG1,2 based on this assumption.

**Results:**

We found differences between AAH and PACG1,2 lesions for some parameters including the number of glands, structures such as the main ductus and basal cells. We found similar properties in the two lesions for the following parameters: localization, multiplicity, diameter of the lesion, focus asymmetry, distance between glands, inflammatory cells in and out of the lesions, secretory cell shape on the luminal side, papillary projection towards the luminal side of gland, the shape of the outer gland, the infiltrative pattern of the gland, glandular pleomorphism, biggest gland diameter and median gland diameter.

**Conclusion:**

We determined that concurrent evaluation of histomorphological features was important to differentiate between AAH and PACG1,2.

## INTRODUCTION

Atypical adenomatous hyperplasia (AAH) is a generally well-defined lesion characterized by the proliferation of small glands in the prostate. The incidence is reported as 1.6-36.9%. They are seen most commonly in the transitional zone.[1–25] The lesion has been named AAH,[1–22] adenosis[23–27] and atypical hyperplasia[28] in various reports. The AAH focus is usually found in the periphery or centre of the hyperplastic nodule.[17] Low magnification shows uniform small glands with a lobular growth pattern. It usually has a prominent and smooth border, but there may be focal irregularities resembling invasion.[18,27,29] The AAH consists of cuboidal or columnar cells with pale or clear cytoplasm. The nucleus is round and located at the basal part of the cell. There is a granular chromatin structure resembling normal prostatic cells. There are often small nucleoli. Basal cells are vaguely defined.[30–32]

Gleason grade 1 prostatic adenocarcinoma (PACG1) is defined as a lesion consisting of monotonous glands of moderate size with minimal stromal invasion, which enlarge by pushing surrounding structures and resemble benign glands with their cytoplasmic features. Gleason grade 2 prostatic adenocarcinoma (PACG2) lesions show infiltrative findings and mild variability in gland size in addition to the features of PACG1.[33,34]

There are many studies on the diagnostic criteria of AAH and its differentiation from adenocarcinoma.[17,35–42] The main criteria arising from these studies are nucleolus size, anti-keratin immunohistochemistry staining (34βE12) specific for basal cells, appearance of the luminal border of the gland and the presence of acidic mucin. However, additional criteria are required when these prove inadequate.

The aim of our study was to assess a total of 18 characteristics consisting of additional structural (glandular, stromal) criteria in addition to the histological criteria for the differentiation of AAH and well-differentiated prostatic adenocarcinoma and to integrate the parameters that can be used to differentiate between these two lesions.

## MATERIALS AND METHODS

### Case selection

We included a total of 105 radical prostatectomy materials evaluated consecutively at the Marmara University, Medical Faculty, Department of Pathology between October 1999 and September 2004. The materials were re-evaluated and those containing AAH or PACG1,2 lesions selected. Three cases consisting of small glands that did not have adequate tissue representing the lesion after the serial sections were excluded from the study and 11 cases of AAH (22 foci) and 15 cases of PACG1,2 (22 foci) were included.

Sections, 4 μm thick, were obtained from the paraffin blocks of the PACG1,2 lesions where the diagnosis was in doubt and all AAH lesions, and the 34βE12 immunohistochemistry stain was used to show basal cells.

## Parameters evaluated

We evaluated the AAH and PACG1,2 lesions by dividing them into four groups according to 18 anatomical and structural parameters. These groups and the parameters are shown in Table 1.

**Table 1:** Grouping of evaluated parameters

Anatomical features	Localizations, multiplicity
Structural and structure-correlated features	
Parameter of lesion features	Diameter of lesion, number of glands, focus asymmetry, lobular patterm, main ductus-like structure, distance between glands, inflammatory cells in-and-out of lesions
Parameter of gland features	Secretory cell shape on luminal side, papillary projection towards the luminal side of the gland, glandular pleomorphism, biggest gland diameter, median diameter of gland
Immunohistochemical features	Existence of basal cells

## Evaluation

### Evaluation of the parameters related to the anatomical features

We determined lesion localization according to proximal or distal placement to the verumontanum. All classifications dividing the prostate into various regions accept the distal section of the verumontanum as PZ.[13,15] All radical prostatectomy material was evaluated following sectioning in our laboratory. The sections were made vertical to the urethra with the apex coded A and the following sections as B, C, D in sequence. We accepted sections A and B of the sections distal to the verumontanum as the peripheral zone.

Multiplicity is the presence of more than one lesion with the same features in a case.

### Evaluation of the parameters related to structural features

Evaluations of the parameters related to structural features are shown in Table 2.

**Table 2:** Evalution of the parameters of the structural and structure-related features

Histological parameters	Evalution			
**Parameters of lesion-related features**				
Diameter of the lesion	Quantitative evaluation with ocular micrometer (μm)			
Number of glands, Quantitative	(1) <10	(2) 10-30	(3) 30-50	(4) >50
Focus asymmetry relative	(1) Smooth lesion circumference	(2) Slightly irregular	(3) Hardly irregular		
Lobular pattern, main ductus-like structure (relative)	(1) (−)	(2) (+)		
Distance between glands relative	(1) No distance between glands	(2) 1-3 stromal cells between glads	(3) >3 stromal cells between glands	
Inflammatory cells in and out of lesions semiquantitative	(1) (−)	(2) Minimal	(3) Many	
**Parameter of the gland features**				
Secretory cell shape on luminal side	(0) Smooth (cytoplasm dimension is equivalent)	(1) Sightly irregular	(2) Very irregular	
Papillary projection towards the luminal side of gland (relative)	(1) Smooth	(2) Glands with slight papillary projection towards the luminal side	(3) Glands with distinct papillary projection	
The form of the outer gland outside (relative)	(1) Smooth	(2) Slightly papillary projection towards the outside	(3) Distinct papillary projection towards the outside	
Infiltrative pattern of gland (relative)	(1) No angulation (gland appearance is round)	(2) Slight angulation in the glands (ellipsoid appearance)	(3) Significant angulation	
Glandular pleomorphism, (relative)	(0) (−)	(1) Mild	(2) Significant	
The biggest gland diameter, median diameter of gland	Quantitative with ocular micrometer (μm)						

We evaluated the secretory cell luminal gland parameter and the irregularity formed at the apical side of the cytoplasm due to the size variability of the cells forming the same gland. This irregularity due to the difference in secretory activity was converted into a numerical value between 0 and 2.

When determining the mean gland diameter, we measured the diameter of all randomly selected glands in an area magnified 20 times and entered the data into the Microsoft Excel 2000 program database to calculate the mean diameter.

### Evaluation of the immunohistochemical features (the presence of basal cells)

We used 34βE12 to show the presence of basal cells in the AAH and PACG1,2 lesions. The lesions were assessed as continuous, focal discontinuous, diffuse discontinuous and none depending on the staining.

## Statistical Evaluation

The data obtained from the study were entered into the database and analyzed with the Statistical Package for Social Sciences for Windows, version 11.0 (SPSS) software program.

The Mann-Whitney *U*-test was used to evaluate the numerical parameters (mean largest gland diameter, mean gland diameter, lesion width and lesion length parameters) between the types.

The χ^2^-test was used to compare the non-numerical values between the types. The glandular shape difference, gland luminal side, gland infiltrative pattern (angulation of gland outer side), number of glands comprising the lesion, the relation of the gland to its surroundings (focus asymmetry), the presence of intralesional and perilesional inflammatory cells, lobular pattern and main ductus parameters were evaluated statistically.

We used Fisher's exact probability test, a subgroup of the χ^2^ relation test, when evaluating the four-celled tables where the expected value was <5 (the distance between the glands).

The results were interpreted at the *P* < 0.05 significance level.

## Immunohistochemical staining method

34βE12 (Keratin, HMW Ab-3 (1/50; Clone 34 β E12; MS-1447-S1; Neomarkers).

We used the streptavidine biotin/horseradish peroxidase (Str.AB/HRP) method to show keratin expression. Ultra V Block (Ultra Vision Kit; TP-125-HL; Lab Vision) drops on the slides were used to prevent nonspecific staining. The tissues were incubated for 10 min in biotinylated secondary antibody (Ultra Vision Kit; TP-125-HL; Lab Vision). Streptavidine Peroxidase (Ultra Vision Kit; TP-125-HL; Lab Vision) was then used. DAB (TA-125-HD, Lab Vision) was used as a chromogen. Cytoplasmic brown staining in basal cells was interpreted as positive.

## RESULTS

The AAH was present at a rate of 10.5% (11 cases) and PACG1,2 at a rate of 15.2% (15 cases) in the 105 radical prostatectomy material were studied [Table 3]. We had 22 cases each of AAH and PACG1,2. It has been found that 63.7% of the AAH lesions (14/22) and 50% of the PACG1,2 lesions (11/22) were localized distal to the verumontanum (A and B sections).

**Table 3:** Anatomical features of the AAH and PACG1,2 lesions

	Data number/Lesion number	Frequency	Localization, VP (%)/VD (%)	Multiplicity (%)
AAH	22. Ka s	10.50%	8 (36.3)/14 (63.4)	4 (36.3)
PACG1,2	15/22	15.20%	11 (50)/11 (50)	4 (26.6)

OS: Data number, LS: Lesion number, Loc: Localization, VP: Proximal to verumontanum, VD: Distal to verumontanum

**Figure 1:**
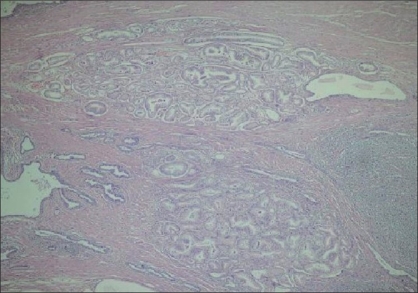
Multiple PACG1,2 lesions (H and E ×40)

Four AAH cases were unifocal (63.7%) and four multifocal (36.3%) (maximum 6 foci). While 11 of the PACG1,2 cases were unifocal (73.4%) and 4 (26.6%) were multifocal (maximum 4 foci) [Figure 1]. The AAH and PACG1,2 were present together in two cases.

There was no significant difference between the multiplicity and frequency parameters of the AAH and PACG1,2 lesions when evaluated with the Mann-Whitney *U*-test (*P* > 0.05).

Our results showed that PACG1,2 lesions consisted of a larger number of glands [Table 4]. This result was found to be statistically significant (*P* = 0.01). We found that this significance was due the higher number of lesions with 10-30 glands in AAH.

**Table 4:** Comparison of the AAH and PACG1,2 lesions in terms of parameters of the lesion features and statistical results

Histological parameters	Evaluation	AAH number (%)	PACG1,2 number (%)	Applied test; *p*	*P*
Lesion diameter	Microns	1306 ± 634 (500-2500 μm)	1710 ± 1197 (500-5000 μm)	Mann-Whitney *U*-test; *P* > 0.05	*P* > 0.05
Lesion dimensions	Micron	2252 ± 1021 (1000-4250 μm)	2715 ± 1380 (1000-6250 μm)	Mann-Whitney *U*-test; *P* > 0.05	*P* > 0.05
Number of glands	Between 10 and 30	6 (27.2)	1 (4.5)	Mann-Whitney	*P* = 0.01
	Between 30 and 50	9 (40.9)	11 (50)	*U*-test; *P* = 0.01	
	> 50	7(31.8)	10(45.4)		
Focus asymmetry	Regular	8(36.3)	9(40.9)	χ^2^-test; *P* > 0.05	*P* > 0.05
(relation of lesions with circuference)	Slightly irregular	9(40.9)	5(22.7)		
	Markedly irregular	5(22.7)	8(36.3)		
	None	9(40.9)	20(90.9)	χ^2^test; *P* > 0.05	*P* = 0.01
	Absent	13(59.1)	2(9.1)		
Main ductus-	Present	3 (13.6)	22(100)	Fisher exact probability	*P* = 0.0001
like structure	Present	19(86.4)	0(0)	test; *P* = 0.0001	
Distance between glands	Absent	15(68.1)	18(81.8)	Fisher exact probability	*P* > 0.05
	Minimal	7(32.8)	4(18.1)	test; *P* > 0.05	
	Marked	0(0)	0(0)		
Inflammatory cells in lesions	Absent	6(27.3)	4(18.1)	χ;^2^ test; *P* > 0.05	
	Minimal	15(68.2)	18(81.9)		
	Many	1(4.5)	0(0)		
Inflammatory cells out of lesions	Absent	0(0)	0(0)	Fisher exact probability	*P* > 0.05
	Minimal	16(72.7)	14(63.6)	test; *P* > 0.05	
	Many	6(27.3)	8(36.4)		

**Figure 2:**
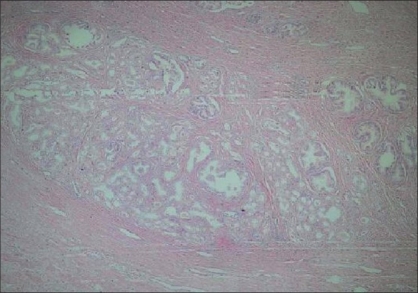
Lobular pattern in AAH (H and E ×40)

**Figure 3:**
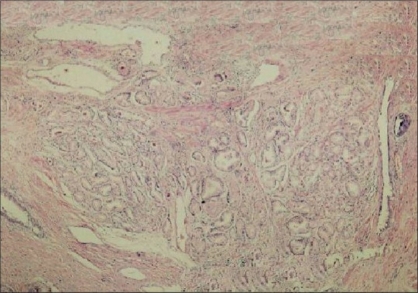
Lobular pattern in PACG1,2 (H and E ×40)

A lobular pattern was present in 59.1% of AAH lesions and 9.1% of PACG1,2 lesions [Figures 2 and 3] and this was found to be statistically significant (*P* = 0.01).

**Figure 4:**
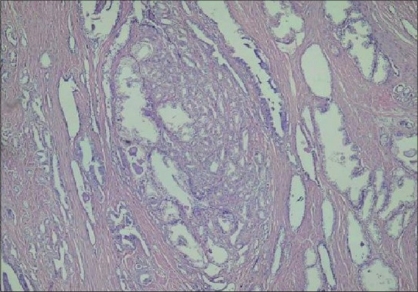
Main ductus-like structure in AAH (H and E ×100)

A main ductus-like structure was present in 86.4% of the AAH cases [Figure 4] but not in the PACG1,2 lesions. This result is statistically very significant (*P* = 0.0001).

The distance between the glands was higher in AAH, but the result was not statistically significant (*P* > 0.05).

**Figure 5:**
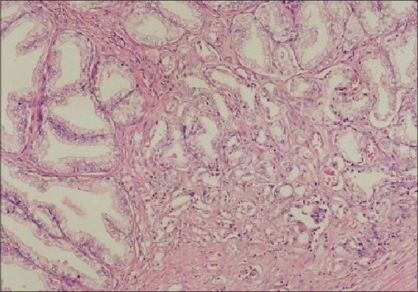
Focus asymmetry in AAH (H and E ×100)

**Figure 6:**
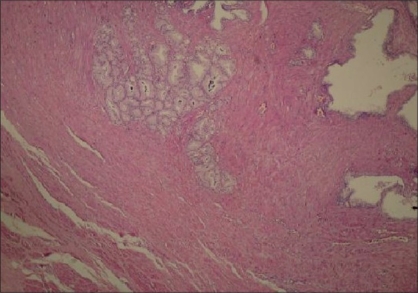
Focus asymmetry in PACG1,2 (H and E ×40)

The lesion size (length, width), the relation of the lesion to its surroundings (focus asymmetry) [Figures 5 and 6] and the presence of intralesional and perilesional cells parameters were similar for the groups and there was no statistically significant difference (*P* > 0.05).

Table 5 presents the comparative glandular features of the AAH and PACG1,2 lesions and the statistical results.

**Table 5:** Comparison of the AAH and PACG1,2 lesions in terms of parameters of the gland features and statistic results

Parameters	Evaluation	AAH, number (%)	PACG1,2 number (%)	Test used
Secretory cell shape on luminal side	Straight (equal cytoptasmic dimensions)	5 (22.7)	9 (40.9)	χ;^2^ test; *P* > 0.05
	Slightly irregular	6 (27.3)	6 (27.3)	
	Markedly irregular	11 (50)	7 (31.8)	
Papillary projection towards the luminal	Absent	5 (22.7)	11 (50)	χ^2^ test; *P* > 0.05
	Minimal projections	14 (63.7)	8 (36.4)	
	Marked projections	3 (13.6)	3 (13.6)	
Shape of the outer of gland	Straight	6 (27.3)	8 (36.4)	χ^2^ test; *P* > 0.05
	Minimal invaginations	3 (59.1)	11 (50)	
	Marked invaginations	3 (13.6)	3 (13.6)	
Infitrative pattern of glands	No angulation	15 (68.1)	8 (36.3)	χ;^2^ test; *P* > 0.05
	Minimal angulation	5 (22.7)	9 (40.9)	
	Marked angulation	2 (9.0)	5 (22.7)	
Glandular pleomorphism	None	1 (4.5)	7 (32.8)	χ;^2^ test; *P* > 0.05
	Mild	7 (32.8)	5 (22.7)	
	Marked	14 (63.6)	10 (45.4)	
Biggest gland diameter	Micron	478 ± 311 (100-1250 μm)	407 ± 169 (140-650 μm)	Mann-Whitney *U*-test; *P* > 0.05
Median diameter of gland	Micron	111 ± 46 (30-225 μm)	117 ± 47 (60-250 μm)	Mann-Whitney *U*-test; *P* > 0.05

**Figure 7:**
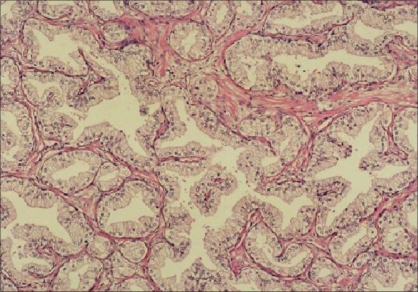
Straight secretory cell luminal side, papillary projection to the gland lumen, glands with irregular exterior in PACG1,2 (H and E ×100)

**Figure 8:**
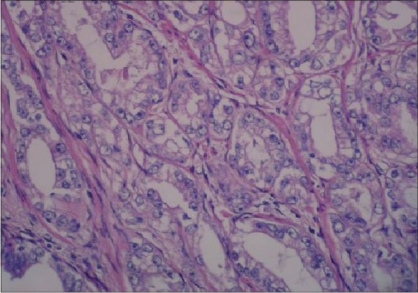
Infiltrative-type glands in PACG1,2 (H and E ×200)

**Figure 9:**
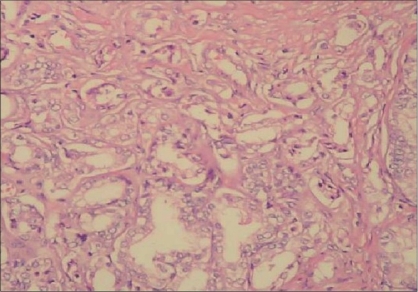
Infiltrative-type glands in AAH (H and E ×200)

The secretory gland luminal side [Figure 7] and an infiltrative-type gland (angulation of gland external side) was seen more often in PACG1,2 lesions but the difference was not statistically significant [Figures 8 and 9] (*P* > 0.05).

Papillary projection into the gland lumen was observed more commonly with AAH but there was no significant difference (*P* > 0.05).

Results for glandular pleomorphism (size variability of glands), mean largest gland diameter, mean gland diameter, shape of gland exterior parameters were similar between the groups with no statistical difference (*P* > 0.05).

**Figure 10:**
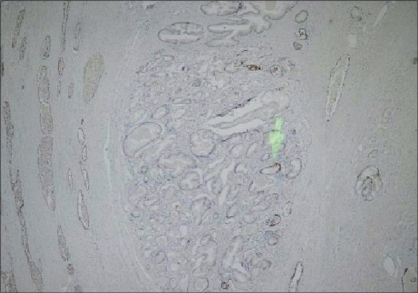
Focal discontinuous staining in basal cells in AAH (34βE12 ×40)

**Figure 11:**
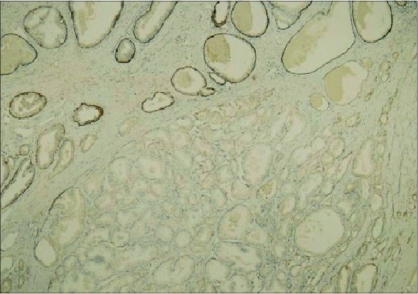
Absence of basal cells in PACG1,2 (34βE12 ×40)

Basal layer cells were present in 50% of AAH lesions in a discontinuous manner and in 50% as diffuse discontinuous. No basal layer cells were observed in PACG1,2 lesions [Figures 10 and 11]. The result was markedly significant (*P* = 0.00001)

## DISCUSSION

A common problem in pathology is evaluating prostatic lesions composed of small acini with suspected malignancy some cancers have been diagnosed as benign lesions while some benign lesions have been diagnosed as cancer especially following needle biopsy due to the inadequacy of the tissue representing the lesion and the absence of histological criteria.[43]

The incidence of AAH varies between 1.6 and 36.9% in reports according to the material studied.[1–25] We did not come across any English articles on the incidence of PACG1,2 lesions. Our values were 10.4% for AAH and 14.3% for PACG1,2. The rates do not reflect the actual incidence as we evaluated the radical prostatectomy material from grade 3 or higher cancers in our study. Studies on autopsy material are therefore needed.

The AAH[5,18,20–21] and PACG1,2[33,34] lesions are seen more frequently in TZ. A localization close to the apex and the periurethral area are reported to be common sites for AAH.[19] We felt that it would not be possible to definitely differentiate between PZ, SZ and TZ in the area proximal to the verumontanum taking the anatomical classifications into account. The minimum frequency in the peripheral zone was 63.4% for AAH and 50% for PACG1,2.

The AAH and PACG1,2 lesions have a tendency to be multifocal.[17,21,23–24,44] This rate changes according to how the material was obtained, but the rate has been reported as 16%[24] in needle biopsies, 68% in TUR material[23] and 46%[17] in prostatectomy material for AAH. Bostwick *et al*. have reported the rate of lesion multifocality as 58% in AAH and 38% in PACG1,2 in their study that also included transurethral resection material.[17] The AAH and PACG1,2 cases were similar for multiplicity in our study with a frequency of 36% for AAH and 26% for, PACG1,2. These rates are consistent with other reports and it is therefore not possible to use multiplicity as a criteria for differentiating between AAH and PACG1,2.

There are only a few studies on the size of the lesions.[19] Bostwick *et al*. have found all foci to be smaller than 11 mm in 17 Gleason grade 1 cancer cases in their study.[19] We found the PACG1,2 lesions to be smaller than AAH lesions, but the difference was not statistically significant.

We did not come across any reports on the number of glands in PACG1,2 and AAH lesions. We determined the number of glands and compared the number between the groups. The number of glands comprising the lesion was markedly higher in PACG1,2 lesions and this result was also statistically significant (*P* < 0.05). This significance was due to the higher number of glands in AAH lesions at 10-30.

There are very few studies on the relation of the lesions with their surroundings (focus asymmetry).[17,23,24,33] Infiltrative features were found at rate of 13-19% in AAH in these studies.[23] Bostwick *et al*. have studied lesions characterized by small glandular proliferation in three categories as AAH, atypical small acinary proliferation and cancer and found no statistically significant difference between the groups although AAH showed less infiltrative features.[17] We found focus asymmetry in 63% of AAH lesions and 59% of PACG1,2 lesions and the difference was not significant. The different results from this limited number of studies may be due to the subjective evaluation.

A lobular pattern is reported as one of the most important characteristics to differentiate AAH and PACG1,2 lesions histologically.[17,23] The AAH shows a lobular pattern[27] while this has not been defined in PACG1,2 lesions. However, we also observed a lobular pattern in 2 PACG1,2 lesions (9.1%). A lobular pattern was present in only 59.1% of AAH lesions although it is reported as a significant characteristic. The lobular pattern carried statistical significance for AAH, but we felt it was not specific.

The presence of a main ductus-like structure is reported as one of the main characteristics of AAH.[17,23] The PACG2 glands are more variable in size than PACG1 lesions. We also felt that the large glands with benign character that could be seen around and sometimes within the lesion could cause a structure similar to the main ductus-like structure seen in AAH. We, therefore, compared AAH and PACG1,2 cases by the appearance of the main ductus-like structure. A main ductus-like structure was present in 86.4% of the AAH cases but not in the PACG1,2 lesions. Similar to other reports, we concluded that the presence of a main ductus was one of the most important characteristics to differentiate between AAH and PACG1,2.

The distance between the glands forming the lesion has been assessed for the first time in our study. Reference information on the presence of intralesional and perilesional inflammatory cells is limited. We concluded that the presence of intralesional and perilesional inflammatory cells could not be used as a criterion for the differentiation of AAH and PACG1,2 lesions.

The straight luminal side of the gland is an important characteristic of high-grade prostatic adenocarcinoma.[45] There are few studies on the subject regarding PACG1,2 lesions.[28,38] Although some authors state that this finding is a feature of PACG1,2 and it is important in the differentiation from AAH,[34] others feel that it is not important in differentiating these lesions.[28,38] When we evaluated the gland luminal side shape in our study, we looked at the shape of the secretory cell luminal side and the papillary projection into the gland lumen features. Secretory cells with a straight luminal side were present in 50% of AAH lesions and the difference was not statistically significant. We decided that the straight luminal cell sides (gland luminal side) that are accepted as an important feature of PACG1,2 and used in the differential diagnosis of AAH and other glandular proliferations are not specific for PACG1,2.

The luminal sides of large glands are more invaginated. Although some AAH lesions consist of large glands mixed with small glands, the majority of PACG1,2 and AAH lesions consist of small glands. McNeal *et al*. have reported that AAH consists of glands with papillary projections while PACG1,2 lesions consist of glands with a straight luminal side.[34] The rate of papillary projection into the gland lumen was higher in PACG1,2 cases (50%) compared to AAH (22.7%), but this difference was not statistically significant.

Our study is the first to assess the outer side of the glands forming the lesions to determine whether they are straight or whether they form projections to the stroma together with the gland infiltrative pattern (angulation of the gland exterior) in AAH and PACG1,2 lesions. Angulation of the external part of the gland was found in 32% of AAH and 64% of PACG1,2 lesions, but this difference was not statistically significant. Our findings indicate that gland features (gland luminal side, gland outer side and gland infiltrative pattern) are not criteria that can be used for the differential diagnosis of PACG1,2 lesions. More studies are needed on the subject.

The PACG1,2 is reported to consist of monotonous glands and AAH of more heterogeneous glands.[27] However, the mean gland size and glandular pleomorphism have not been found useful in other studies in differentiating AAH from adenocarcinoma.[28,38] Montironi *et al*. have found mild shape variability in 25 and 75% of the glands in AAH and cancer and marked shape variability in 75 and 25%, respectively.[27] Glandular pleomorphism was found in 95% of AAH lesions and 68% of PACG1,2 lesions, but there was no statistical significance.

Our study is the first to evaluate the largest gland diameter and the mean gland diameter in a comparative way. There is one previous study where the gland diameter was measured in PACG1,2 lesions.[46] The largest gland diameter was in AAH in our study while the mean gland diameter was larger in PACG1,2 lesions. The results are similar with no statistical significance. We felt that these criteria could not be used in the differential diagnosis of AAH and PACG1,2 lesions.

Another important criterion in the differentiation of AAH and PACG1,2 lesions is the presence of basal cells. Staining of basal cells with 34βE12 enables differentiating many lesions. Cells staining specifically with 34βE12 are not present in cancers and are seen characteristically in an interrupted manner in AAH.[30–33,35–36,38–42] A few studies have reported a positive immunereaction of neoplastic cells with 34βE12.[22,27,30,40,42] However, it has been reported that the antigen recovery method may be responsible for the positive staining.[47,48] Another study has reported that the few cells staining positively with 34βE12 did not morphologically resemble basal cells at all.[42] All basal cells of the normal prostatic gland show strong immunoreactivity with 34βE12, but there may be disturbed staining continuity in inflammation.[17] Formalin fixation and heat damage can also cause false negativity in basal cells[23] and the absence of staining in some basal cells while others are stained, especially in needle biopsies and other biopsies where the material is small makes the diagnosis difficult. We confirmed this morphological finding with 34βE12 and observed the presence of basal cells in all AAH lesions in the gland periphery while there was no positively staining cell in PACG1,2 lesions. Positively staining basal cells were present in a focal manner in 50% of AAH lesions and diffuse in 50%.

In conclusion, the absence of basal cells in PACG1,2 and their occasional presence in AAH was accepted as the most important diagnostic criterion. A lobular pattern, stated as one of the most important features in histologically differentiating AAH and PACG1,[2] was significantly higher in AAH lesions but was also found, albeit at a lower rate in PACG1,2 lesions. Although it is reported that a gland with a straight luminal side is important for PACG1,2 lesions, we did not find a significant difference between AAH and PACG1,2 lesions.

Although a lobular pattern and the presence of basal cells are important parameters for the differentiation of AAH and PACG1,2 lesions, the differential diagnosis continues to be difficult in some cases, especially with needle biopsy material where a limited number of criteria can be evaluated. We feel that it is important to determine the histological parameters together in AAH lesions where basal cells are focal or few in numbers. Our results suggest that the combination of architectural and immunohistochemical features allows identification of AAH and accurate differentiation from PACG1,2 in most cases.
